# Mastering movement: A Cross-sectional investigation of motor competence in children and adolescents engaged in sports

**DOI:** 10.1371/journal.pone.0304524

**Published:** 2024-05-30

**Authors:** Fábio Saraiva Flôres, Denise Paschoal Soares, Renata M. Willig, Ana Carolina Reyes, Ana Filipa Silva

**Affiliations:** 1 Insight: Piaget Research Center for Ecological Human Development, Piaget Institute, Almada, Portugal; 2 Research Center in Sports Performance, Recreation, Innovation and Technology (SPRINT), Melgaço, Portugal; 3 Liberal Arts Department, American University of the Middle East, Kuwait City, Kuwait; 4 Escola Superior Desporto e Lazer, Instituto Politécnico de Viana do Castelo, Viana do Castelo, Portugal; Instituto Politecnico de Viana do Castelo, PORTUGAL

## Abstract

**Introduction:**

Motor Competence (MC) is related to the development of healthy lifestyles in children and adolescents, and many studies have compared it with different variables, including sports participation. This investigation aimed to characterize the components and total scores of MC regarding different physical activity practices, such as physical education (PE) classes, futsal, volleyball, and ballet, and to compare MC regarding sports, sex, and age-groups.

**Methods:**

Using a cross-sectional study, 398 Portuguese children and adolescents (398 participants: 200 boys and 198 girls; 12.649±3.46 years) were conveniently chosen between 2022 and 2023. Four groups of different sports were created: PE classes (n = 187), futsal (n = 80), volleyball (n = 101) and ballet (n = 30), and four different age groups: 7 to 9 years old (n = 117); 10 to 12 years old (n = 65), 13 to 15 years old (n = 96) and, 16 to 19 years old (n = 120). All participants reported to participate in at least two training sessions per week (1 hour each) for at least two years. MC was assessed with Motor Competence Assessment (MCA) comprising three components with six tests (two tests for each component). Participants’ total MC was calculated as the average of the three components of the MCA. Data were analyzed by applying ANOVA one-way with LSD post-hoc.

**Results:**

Differences were found in MC among groups, where futsal participants showed better scores in general. Sex comparison showed that boys have a higher MC when practicing futsal, especially compared to volleyball players. Age-group analysis showed that younger participants had better MC levels compared to older ones.

**Conclusion:**

The whole group showed the 50th percentile of MC, but volleyball adolescents showed results below this reference. MC is intrinsically linked to an individual’s motor experiences and motivations rather than depending directly on the specific practice of the sport.

## Introduction

It is well established that when children grow, they become influenced by an extensive ecological framework—represented by Bronfenbrenner’s mesosystem [1, 2]. This mesosystem comprises multiple microsystems (immediate contexts) that children attend daily. Therefore, contexts such as the home, the neighborhood, the school, and sports activities start to be part of children’s lives when they enter the fundamental movement phase [3].

Every microsystem is composed of different affordances that shape motor behavior [4] and, consequently, each context has objects, places, surfaces, events, and people that provide different opportunities to move. Koller [5] pointed out that some microsystems can provide richer affordances compared to others, and thus have greater potential for promoting better levels of development, learning, and competence. For instance, physical education (PE) classes aim to promote physical competence, develop the children and adolescents’ movement efficiently, effectively, and safely, and understand what they are doing, in other words, to achieve and develop physical literacy [6]. Although, in Portugal, PE bases its classes on the learning of the specialized movements of each sport, it has a more holistic view of movement development, immediately including more movements from different sports and not just replicating specialized movements of a single sport in a competitive model.

The literature has shown that well-structured microsystems (i.e., sports activities) seem to enable the development and acquisition of children’s fundamental motor skills [7], which is related to the Motor Competence (MC) concept. MC can be understood as the mastery of fundamental motor skills, being the basis for specialized motor skills development throughout lifespan [8, 9]. Therefore, MC is a global term related to movement development and performance and has been understood as a person’s ability to be proficient in locomotor, stability, and manipulative gross motor skills [10]. Furthermore, Rodrigues et al. [11] highlight that stability, locomotor, and manipulative skills change through known periods of initial learning patterns, to elementary phases of practice, and finally to the achievement of an advanced motor pattern, which can be strengthened over exercise and practice to a specialized (sportive) motor skill.

The literature explains that MC enables children and adolescents to participate in various physical activities and physically active play [12, 13]. Barnett et al. [14] found that better levels of MC can predict children’s subsequent physical activity levels, and Campos et al. [15] suggested that enhancing locomotor skills could increase the range and diversity of social and cognitive experiences, thereby promoting their development during infancy. Since the regular environments, physical activity and motor development are tightly interwoven during childhood and adolescence [16], some research has been conducted regarding sports participation [17–20].

Sports participation can be understood as the engagement in organized sessions related to a specific sport, which can provide different opportunities to be physically active as well as favor psychosocial development and the acquisition of life skills [20, 21]. González-Víllora et al. [21] also explain that physical and physiological performance are significantly higher during sports practice. The literature also points out that sports permit children and adolescents to meet situation-specific demands in the culture of sports and exercise [22, 23]. Therefore, Ferreira et al. [17] examine the association between sports participation and MC in schoolchildren and the results indicate that consistent participation in sports was associated with high scores of MC. In addition, children with higher levels of MC were more involved in sports activities than children with lower MC [18]. De Meester et al. [24] found that adolescents with low MC, but high perceived MC were more active compared to those with low MC who accurately estimated themselves. Authors highlight that developing perceived MC among adolescents with low levels of MC seems important to stimulate engagement in physical activities and sports participation. It is also important to mention that boys seem to receive more opportunities to practice PA, especially in ball sports, while girls have lower levels of PA and receive more opportunities to practice individual sports, such as dancing [22].

In the last years, new instruments have been developed to analyze and assess MC during childhood and adolescence. One of the most prominent is the Motor Competence Assessment (MCA), developed in Portugal [25]. Since its creation, the MCA model and normative values of this innovative product-oriented instrument were established from the age of 3-to-23 years old [11, 25–27]. Luz and colleagues established the construct validity using a model with three correlated components (locomotor, stability, and manipulative), without a marked developmental ceiling effect, and of feasible execution to diminish observation errors [11]. The Portuguese normative values for the MCA were published [26], differentiating participants according to sex and age. Since then, some studies have reported the MC of the Portuguese population, indicating that children and adolescents typically demonstrate values between the 40th and 50th percentiles, meaning they exhibit below-average levels of MC [9, 28]. Additionally, COVID-19 has resulted in a decline in MC values among Portuguese children and adolescents [28, 29].

Some past investigations showed that MC is associated with the development of healthy lifestyles [12, 13, 30], can be a significant predictor of metabolic syndrome in children and adolescents [31], is associated with flexibility in adolescents [32], is reliable to assess youth volleyball players MC [33], and can influence the perceived exertion in young adults [34]. Researchers also compared MC regarding their country [35, 36], sex [37, 38], and the quality of their environments and affordances provided [39]. Different reviews are showing that MC is a complex biocultural construct and, its investigation requires a transdisciplinary approach [40, 41]. For instance, in a cross-cultural study comparing the motor competence of children with a mean age of 10, a study found that Portuguese children, regardless of sex, demonstrated higher proficiency in locomotor activities than the U.S. In contrast, U.S. children showed better performance than Portuguese children in throwing activities [36]. Also, with a lower mean age (7.81 ± 1.50 years), Flôres et al [42], found that southern Brazilian boys performed significantly better than the normative values of Portuguese boys in locomotor and manipulative tasks, with no differences found in stability tasks. In contrast, southern Brazilian girls demonstrated lower performance compared to Portuguese girls.

Hence, the literature has suggested that a larger range of movements can support physical activity engagement across the lifespan [43], and regular physical activity could promote the development of MC, which would lead to long-term participation in different activities and sport participation. Therefore, investigating and characterizing how sports environments influence children and adolescents’ MC continues to be of paramount importance. As far as we know, no other investigation has assessed children and adolescents’ MC regarding their participation in different sports. Thus, the purpose of this investigation was to characterize the components and total scores of MC regarding different physical activity practices, such as PE classes, futsal, volleyball, and ballet, and to compare MC regarding sports and sex. As a second goal, we also perform a comparison between age groups. It was hypothesized that children and adolescents will present different levels of MC, but sports participation will outperform the ones practicing only in PE classes. In addition, it was also expected that boys would outperform girls, and older participants would present higher MC levels.

## Methods

### Participants

The present investigation is a cross-sectional study, and all participants were chosen by convenience in Portugal between 2022 and 2023. The sample size was calculated using the GPower v 3.1.9.7 software [44] using the following parameters: Cohen’s effect size of 0.25 for One-way ANOVA, error probability α = 0.05, and β = 0.99, resulting in a sample of 385 participants. Therefore, 398 participants (200 boys and 198 girls; 12.649±3.46 years; 19.83±4.03 kg/m^2^) were recruited in different regions of Portugal.

The inclusion criteria were as follows: absence of injuries or illness within the last four consecutive weeks; and previous experiences in sports (apart from school-only PE participant). None of the participants had any developmental difficulties or medical conditions to perform the test. As an exclusion criterion, in addition to having any injury or illness, not having the informed consent signed by the guardian was also considered.

Participants were grouped into different groups (see sample characteristics in Table 1): futsal (n = 80; 36 boys and 44 girls), volleyball (n = 101; 46 boys and 55 girls), and ballet (n = 30 girls). The PE classes were treated as a control group (n = 187; 118 boys and 69 girls).

**Table 1:** Sample characteristics by sport.

Group	Sex	Variables	N	Mean	SD
Futsal	Boys	Age (y)	36	8.58	1.13
		Height (m)	36	1.27	0.06
		Weight (kg)	36	26.71	6.28
		Body Mass Index (kg/m^2^)	36	16.49	3.80
	Girls	Age (y)	44	15.52	2.85
		Height (m)	44	1.61	0.15
		Weight (kg)	44	59.05	14.68
		Body Mass Index (kg/m^2^)	44	22.09	3.47
PE classes	Boys	Age (y)	118	11.73	3.18
		Height (m)	118	1.43	0.16
		Weight (kg)	118	40.96	15.94
		Body Mass Index (kg/m^2^)	118	19.15	3.97
	Girls	Age (y)	69	10.03	2.00
		Height (m)	69	1.34	0.10
		Weight (kg)	69	32.59	10.02
		Body Mass Index (kg/m^2^)	69	17.72	3.62
Ballet	Boys*	Age (y)	0	-	-
		Height (m)	0	-	-
		Weight (kg)	0	-	-
		Body Mass Index (kg/m^2^)	0	-	-
	Girls	Age (y)	30	12.67	2.77
		Height (m)	30	1.48	0.14
		Weight (kg)	30	44.85	15.15
		Body Mass Index (kg/m^2^)	30	19.82	4.15
Volleyball	Boys	Age (y)	46	15.85	1.38
		Height (m)	46	1.64	0.07
		Weight (kg)	46	60.62	10.42
		Body Mass Index (kg/m^2^)	46	22.47	2.48
	Girls	Age (y)	55	15.49	1.60
		Height (m)	55	1.62	0.08
		Weight (kg)	55	58.35	9.62
		Body Mass Index (kg/m^2^)	55	22.08	2.51

*No boys practicing ballet were recruited for this sample.

Participants were also grouped into four different age groups: 7 to 9 years old (n = 117); 10 to 12 years old (n = 65), 13 to 15 years old (n = 96), and 16 to 19 years old (n = 120). More details about the sample characteristics can be seen in Table 2.

**Table 2:** Sample characteristics by age group.

Sex	Age-group	Variables	N	Mean	SD
Boys	7 to 9 years	Age (y)	73	8.33	0.65
		Height (m)		1.26	0.04
		Weight (kg)		25.98	6.14
		Body Mass Index (kg/m^2^)		16.35	3.74
	10 to 12 years	Age (y)	32	11.00	0.98
		Height (m)		1.40	0.06
		Weight (kg)		35.88	6.68
		Body Mass Index (kg/m^2^)		18.32	2.62
	13 to 15 years	Age (y)	44	13.82	0.76
		Height (m)		1.54	0.04
		Weight (kg)		49.80	6.67
		Body Mass Index (kg/m^2^)		20.96	2.41
	16 to 19 years	Age (y)	51	16.75	0.77
		Height (m)		1.68	0.05
		Weight (kg)		65.63	8.26
		Body Mass Index (kg/m^2^)		23.25	2.55
Girls	7 to 9 years	Age (y)	44	8.25	0.58
		Height (m)		1.26	0.04
		Weight (kg)		24.66	4.95
		Body Mass Index (kg/m^2^)		15.58	2.95
	10 to 12 years	Age (y)	33	11.06	0.90
		Height (m)		1.40	0.05
		Weight (kg)		36.01	6.21
		Body Mass Index (kg/m^2^)		18.47	3.01
	13 to 15 years	Age (y)	52	14.00	0.79
		Height (m)		1.54	0.05
		Weight (kg)		51.37	6.87
		Body Mass Index (kg/m^2^)		21.69	2.91
	16 to 19 years	Age (y)	69	16.68	0.76
		Height (m)		1.68	0.04
		Weight (kg)		64.59	6.29
		Body Mass Index (kg/m^2^)		22.91	2.01

All participants reported to participate in at least two training sessions per week (1 hour each) for at least two years (futsal, volleyball, and ballet groups). Furthermore, PE classes lasted between 60 and 120 minutes per class and were attended twice a week by all participants. Finally, the children and adolescents in the PE classes group did only school PE and no other regular sports activities. The other participants (from the other three groups) participated regularly in PE classes and training in their respective sports.

### Instruments and procedures

This investigation was conducted on regular training days, before practice, and after 24 hours of rest. The assessments were performed in a controlled place during the evenings (testing always took place in a gymnasium), with an environmental temperature of 21.5 degrees Celsius and relative humidity between 80%. Data were collected between January and June 2023.

Before data collection, all participants completed a 10-minute warm-up, as they normally do in their practice. The test set was structured to be developed in groups of five participants per task and was administered by trained examiners. A verbal explanation and a proficient demonstration of all tests were provided. A test trial was provided to all participants before the test administration began, and all participants received the instruction that tests should be performed at their maximum. No feedback regarding the test results or skill performance was provided. All data collection was supervised by one of the authors of this study.

Participants were evaluated using the MCA [11, 25–27]. The MCA comprises six tests and three components: (1) Locomotor—Standing Long Jump (SLJ) and the Shuttle Run (SHR) tests; (2) Stability—Shifting Platforms (SP) and the Jumping Sideways (JS) tests; (3) Manipulative—Ball Kicking Velocity (BKV) and Ball Throwing Velocity (BTV) tests. The MCA was administered following the protocol proposed in the literature [11, 25–27]. The reliability and validity data are also provided [27], showing perfect values (0.999 to 1.000) for all models, and all tests are quantitative (product-oriented) without a marked developmental ceiling effect. To ensure valid data collection and avoid the learning effect, three repetitions of each test were performed, with the first being characterized as familiarization and the subsequent two as the effective application of the test, as suggested by Silva et al. [45]. Normative values based on sex and age can be assigned to each test, component, and MCA total score [26]. The tests and the MCA scoring method are described below.

### Motor competence assessment

#### Locomotor component

*Standing long jump*. In the SLJ test, participants executed the jump with maximal effort, starting with both feet aligned together. The distance was assessed by the difference between the starting point and the position of the heel of the foot closest to the starting point following the jump (measured in centimeters). Participants performed three attempts, and the best were used for data analysis.

*Shuttle run*. During the 4x10m Shuttle Run, all participants ran at maximal speed toward a line placed 10 meters apart, picked up a wooden block, ran back, and placed it beyond the starting line. Then, the participants needed to run back to retrieve the second wooden block and carry it back across the finish line. The final score was the best time of the two trials.

#### Stability component

*Shifting platforms*. Participants moved laterally for 20 seconds while using a pair of wooden platforms (25cm x 25cm x 2cm). Every successful transfer from one platform to the other was scored with two points (one point for each step–passing the platform and moving the body to the platform). The best of two trials was considered.

*Jumping sideways*. In the JS, participants performed a sideways jump over a wooden beam (60 cm length × 4 cm height × 2 cm width). Participants should keep both feet together while jumping as fast as possible for 15 seconds. Each correct jump scored one point, and the best result over two trials was considered.

#### Manipulative component

*Ball kicking velocity*. The BKV required participants to kick a soccer ball (circumference, 64.0 cm; mass, 360.0g) against a wall with maximum effort. The speed of each kick was quantified using a radar gun (Pro II STALKER radar gun) (measured in meters per second). It used the fastest speed of the three kicks.

*Ball throwing velocity*. The BTV required participants to use an overarm action to throw a size tennis ball (diameter, 6.5cm; mass, 57.0g) against a wall with maximum effort. The speed of each throw was quantified in meters per second using a radar gun (Pro II STALKER radar gun). The fastest speed of three throws was used.

#### MCA calculation and total score

Individual scores were transformed into age- and sex-related percentiles using the Portuguese normative values of the MCA [26]. The stability, locomotor, and manipulative components were calculated using the average of the respective two tests’ percentile positions. Therefore, the participants’ total MC was calculated as the average of the three MCA components.

Oral consent was obtained from the participants, and written consent from their legal guardians before the assessment. In addition, participants over 18 years old also signed the written consent form. This research was approved by the University Ethics Committee (ISEIT de Almada, Instituto Piaget, Portugal, P02-S09-27/04/2022), and the study protocol followed the Declaration of Helsinki guidelines [46].

### Statistical analysis

Descriptive analysis with mean and standard deviation was used to characterize data. The Kolmogorov-Smirnov test confirms data normality. To compare different sports and age groups, the ANOVA one-way was used with LSD post-hoc. To compare between sexes, an independent t-test was applied. The Statistical Package for Social Sciences (SPSS), version 29.0, was used, adopting an alpha level of significance of 5%.

## Results

General results showed that boys and girls had similar MC scores (F (1) = 0.712; p = 0.40, η^2^ = 0.00). Nonetheless, boys outperformed girls in the stability component (F (1) = 8.747; p = 0.00, η^2^ = 0.02).

Concerning locomotor component, Table 3 shows that no significant differences were found between sports and PE classes. Regarding stability component the only significant difference found was between volleyball and PE classes (p = 0.04). In addition, volleyball athletes were outperformed by PE classes students (p = 0.02) and futsal players (p = 0.01) in the manipulative component of the MC. Our data also showed some interesting significant differences between groups and sex (Table 3). Regarding futsal, boys outperformed girls in all categories: locomotor (F (1) = 16.30; p = 0.00, η^2^ = 0.17), stability (F (1) = 32.90; p = 0.00, η^2^ = 0.29), manipulative (F (1) = 4109.30; p = 0.00, η^2^ = 0.58), and MC (F (1) = 71.32; p = 0.00, η^2^ = 0.48). Concerning volleyball, girls showed higher levels of locomotor (F (1) = 27.34; p = 0.00, η^2^ = 0.22), stability (F (1) = 4.04; p = 0.05, η^2^ = 0.04), manipulative (F (1) = 20.30; p = 0.00, η^2^ = 0.17), and MC (F (1) = 26.61; p = 0.00, η^2^ = 0.21) compared to boys. In PE classes, the differences were not significant differences between boys and girls, with locomotor (F (1) = 1.56; p = 0.21, η^2^ = 0.01), stability (F (1) = 2.05; p = 0.15, η^2^ = 0.01), manipulative (F (1) = 0.84; p = 0.36, η^2^ = 0.01), and MC (F (1) = 0.14; p = 0.707, η^2^ = 0.00).

**Table 3:** Mean and standard deviation of the percentile of MC and each component for the different groups and sex.

Sex	Groups	Locomotor (%)		Stability (%)		Manipulative (%)		Motor Competence (%)	
		Mean	SD	Mean	SD	Mean	SD	Mean	SD
Boys	Physical Education	58,67	27,50	45,80	24,69	48,87	28,40	51,11	22,32
	Futsal	70,78^a^	18,95	57,33^a^	19,00	86,43^a^	13,40	71,51^a^	11,57
	Volleyball	53,27	19,67	32,96	22,76	30,01	25,30	38,75	15,89
Girls	Physical Education	63,81	26,52	40,58	22,95	52,64	24,68	52,34	20,22
	Futsal	51,00	23,88	30,79	21,79	33,62	27,75	38,47	20,99
	Volleyball	73,20^b^	18,57	42,05^b^	22,56	52,17^b^	24,04	51,87^b^	20,00
	Ballet	66,56	24,69	34,78	20,90	52,03	29,49	51,87	20,00
Boys + Girls	Physical Education	60,57	27,19	43,87^c^	24,13	50,26^c^	27,08	51,57	21,52
	Futsal	59,90	23,82	42,73	24,39	57,38^d^	34,60	53,34	23,93
	Volleyball	64,12	21,44	37,91	22,99	42,08	26,89	48,04	18,55
	Ballet	66,56	24,69	34,78	20,90	52,03	29,49	51,87	20,00

^a^ Boys significantly outperform girls

^b^ Girls significantly outperform boys

^c^ PE classes outperformed volleyball

^d^ Futsal outperformed volleyball

*p<0.05.

**Fig 1:**
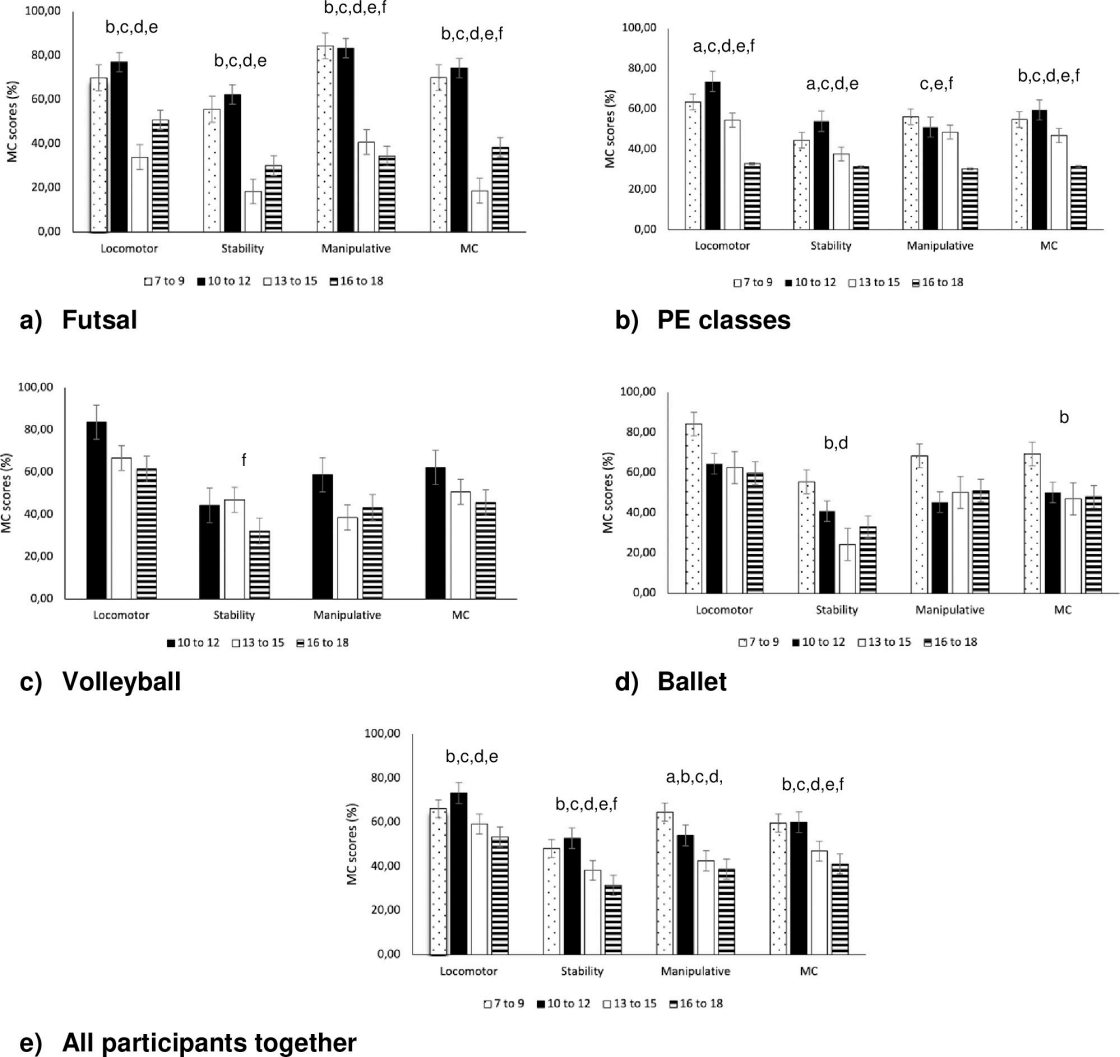
Comparison of the percentile of MC among age groups for the different sports groups. ^a^ difference from 7- to 10-year-old group to 10 to 12; ^b^ difference from 7- to 10-year-old group to 13 to 15; ^c^ difference from 7- to 10-year-old group to 16 to 18; ^d^ difference from 10- to 12-year-old group to 13 to 15; ^e^ difference from 10- to 12-year-old group to 16 to 18; ^f^ difference from 13- to 15-year-old group to 16 to 18; *p<0.05.

When comparing MC among groups practicing different sports but stratified by age group (Fig 1), the results showed that there were significant differences between age groups in all groups. In Futsal, significant differences in MC were found between almost all age groups. The comparison between the 7 to 9 and 13 to 15 age groups showed significant differences (p = 0.00), indicating that the 13 to 15 age group had significantly lower MC scores compared to the younger group. This pattern was consistent when the 7 to 9 age group was compared with the 16 to 18 age group (p = 0.00), again showing higher levels of MC than the older age group. When comparing the 10 to 12 age group with the 13 to 15 age group (p = 0.00), and the 16 to 18 age group (p = 0.00), there were significant decreases in MC.

In the PE classes group, results also indicated significant differences. Contrary to what was found in futsal participants, when comparing the 7 to 9 and 10 to 12 age groups, the older children showed better MC scores (p = 0.02). Differences were also found when the 7 to 9 age group was compared to the 16 to 18 age group (p = 0.00), showing a decrease in MC levels through age. The 10 to 12 age group compared to the 13 to 15 (p = 0.00), and 16 to 18 age groups (p = 0.00) also showed significant decreases, demonstrating that in PE classes, older students tend to have lower levels of MC.

For the Ballet group, no significant differences were observed, indicating that within this group, age did not seem to play a significant role in the development of MC. In the Volleyball group, although there were no significant results regarding MC, comparisons did reveal a trend towards significance comparing the 10 to 12 and the 16 to 18 age group (p = 0.08), suggesting potential decreases in MC with age. Finally, when all participants were analyzed together, younger children showed better MC levels compared to the older ones.

## Discussion

The present study aimed to characterize MC and its components considering different sports experiences in Portuguese children and adolescents. In general, it was possible to notice that MC percentiles ranged between the 40^th^ and 50^th^, which is consistent with previous studies conducted on the Portuguese population [9, 28]. Considering the whole sample, no differences in MC were found between boys and girls, except in the stability component, where boys outperformed girls. Regarding the analysis by age, it was found significant differences between age groups in all groups, with the younger children achieving higher MC levels compared to the older ones. An intriguing finding is that athletes (from various sports) did not demonstrate significantly better MC compared to the group that only participated in PE classes. In fact, volleyball players showed the lowest MC values of our sample. Considering all together, and especially the fact that athletes did not present better MC percentiles as expected, those results seem to suggest that MC is closely dependent on motor experiences and motivations and not directly on the sports practice, sex, or age [47].

A potential reason for the lower results of MC in volleyball could be related to the sport’s emphasis on specific and intricate movements like the bump, which may not align closely with the movement patterns assessed in the MC tests. In contrast to futsal, which showed better MC results, volleyball lacks common childhood skills such as kicking. While one might assume that actions like setting or serving could be analogous to a throw evaluated in the MCA, neither setting uses one hand only, nor does serving resemble the type of throw being evaluated. Thus, the role of experience is significant, since children develop familiarity through repeated engagement in activities, which enables quicker task execution and multitasking capabilities [48, 49]. In fact, a deeper analysis of the nervous system and its connection with the muscular system, tells us that the formation of fatty myelin around the axons of neurons in children leads to enhanced cognitive processing characterized by increased speed, coordination, and complexity. Myelination facilitates efficient communication between neurons, enabling the execution of coordinated behaviors [49, 50]. Therefore, the combined experiences performed by children and adolescents may play a more influential role in their motor skill development than simply engaging in sports.

Regarding the comparison between sexes, the lowest MC values were registered by boys’ volleyball players and girls’ futsal players. Girls’ futsal players demonstrated the least favorable outcomes in our sample, exhibiting a clear disadvantage when compared to their counterparts in the same sport in all MC components. Nevertheless, girls significantly outperformed boys’ volleyball players in all MC components. Despite those differences in futsal and volleyball, when analyzing boys and girls who attend exclusively PE classes presented similar results in all tests. Our results also showed that boys and girls in our sample presented no significant differences in MC. Although most of the studies reported that boys outperformed girls in all MC components [26, 51], some others showed better results in locomotor abilities in girls [52, 53]. The differences found between sexes are usually explained by social factors, where it is suggested that boys are more encouraged to participate in sports than girls [54, 55]. Nevertheless, most of those studies were conducted with primary school or kindergarten children, reflecting a lack of studies involving adolescents. It should be noted that, in the present study, all the percentiles calculated were normalized to age and sex using the Portuguese normative values [26].

When comparing the different age groups, it was evident that the younger age groups stand out in comparison to the adolescent ages. This result did not align with a systematic review and meta-analysis that demonstrated a correlation between age and children’s gross motor competence [56]. Nevertheless, another study that registered declines in MC in girls, proposed that it was linked to reduced opportunities for physical activity, as evidenced by the study’s finding that girls’ physical activity levels declined [57]. Indeed, motor development in young children during the earliest years is primarily influenced by biological maturation. However, as children mature, their motor development is increasingly shaped by practice and the opportunities they must engage in physical activities. As a result, the relationship between age and gross motor competence is expected to change across the developmental stages of early childhood, preschool, childhood, and adolescence [56]. Moreover, the significant reduction in opportunities over recent years due to the COVID-19 pandemic has led to a decline in MC values among Portuguese children and adolescents [28, 29], which could also have interfered with our findings.

The results found in the present study regarding the MC levels (only around percentile 50 or less, even among athletes) are consistent with recent research in young children. The previous research has indicated a decline in MC levels over the past few decades [58, 59]. These outcomes raise concerns about the prospects of the upcoming generation of sports athletes, as a body of literature has highlighted a correlation between fundamental movement skills, as evaluated through MC assessment, and engagement in sports [60–62]. Those authors argued that greater proficiency in fundamental movement skills could facilitate the acquisition of more complex motor skills due to a strong foundational movement pattern, while subject to refinement through practice, can be adapted to novel sports contexts [58]. Expanding on this idea, Vandorpe et al. [20] have even speculated that MC in 6- to 8-year-old children predicts future sports participation. Considering that the development of fundamental movement skills competence is not an innate process [58, 63], it becomes essential to provide children with appropriate and tailored opportunities for practice. This approach is necessary for them to acquire and enhance multiple movement skills effectively, which subsequently lays the groundwork for the mastery of more advanced sports skills [64].

Contrary to the existing literature suggesting a relationship between MC levels and engagement in sports, when analyzing all children together, the current study does not provide support for this notion. This discrepancy raises uncertainty about whether these athletes, particularly the volleyball players, who exhibited lower MC levels and their coaches truly perceive these skills as "fundamental". Indeed, the process of skill development and acquisition is intertwined with various social, cultural, and psychological influences [65]. Consequently, distinct groups exhibiting varying sports preferences might still be driven by common motivations for skill practice and training, which could account for the disparities observed in stability and manipulative capabilities.

An additional question that arose within the scope of this investigation pertains to the possibility that experience, exemplified by exposure to diverse contexts, could exert a more substantial influence on MC than mere engagement in a particular sport. The progression towards more intricate coordination patterns has been demonstrated to unfold over extended periods, possibly spanning several hours or even years of deliberate practice [58, 64]. However, the mere replication of specific techniques and movements within sports clubs has the potential to constrain the acquisition of novel skills. Hence, if early specialization is practiced in clubs, it could result in athletes achieving a high degree of expertise in particular movements but lacking the variability to experiment with new actions within diverse settings. Therefore, the extent to which a club promotes an athlete’s long-term development could significantly impact the evolution of their MC. Therefore, forthcoming investigations should consider critical learning aspects associated with practice regiments.

As mentioned earlier, the literature suggests that learning skills within diverse contexts, like game-based activities, are more effective than learning them in isolation [66]. This is due to the focus on overcoming movement challenges rather than just the form of execution. From this perspective, children and adolescents not only acquire skills, but also face challenges, discover movement possibilities (affordances), and learn how to select effective solutions [66]. This view aligns with the concept of Physical Literacy as initially proposed by Whitehead [47]. Therefore, the crucial time for every child to access an enriching environment for discovering their abilities is during PE classes. This is particularly important because childhood (<11 years in girls and <13 years in boys) [67] is characterized by heightened neural plasticity, optimal for developing motor skills and athletic competency [68]. However, even with the accelerated development of the nervous system, refining movement skills is limited without consistent practice, with or without guidance from a qualified coach [69].

Lastly, another inquiry arises within the scope of this study: Are we genuinely evaluating MC? The literature lacks a unanimous consensus concerning the appropriate methodologies for evaluating and documenting MC. Multiple assessment batteries contend that they gauge fundamental motor skills, potentially allowing for extrapolations about comprehensive MC [70]. Indeed, the movement repertoire encompassed by the MC battery employed is defined in the literature to evaluate fundamental motor skills, which are defined as essential basic learned movement patterns not naturally occurring but foundational for more intricate physical and sporting activities. These fundamental motor skills can be broadly categorized into locomotor (pertaining to body movements like running), object control (manipulative skills like ball-catching), and stability skills, including balancing [71]. Nonetheless, a pivotal aspect of learning pertains to the intentionality intertwined with specific perception-action couplings [72, 73]. This implies that the notion of a universally "fundamental" movement is debatable, as the significance and assessment of a movement are inherently entwined with the intention underlying its execution [66]. Therefore, excelling in a sport transcends mere performance enhancements in the standardized tests designed for evaluating the considered fundamental motor skills.

The present study has some pitfalls that must be addressed. Firstly, the overall physical activity of the participants was not analyzed, only sports were considered for comparison. This was because the authors focused exclusively on structured practices. Another limitation was the absence of data for male participants in ballet. However, ballet is much more commonly practiced by girls than boys, at least in Portugal.

## Conclusion

While the current study has evoked more inquiries than resolutions, some insights can be gathered. It seems that the overall MC level among athletes and non-athletes remains around the 50th percentile, with volleyball athletes exhibiting values below this benchmark. Nevertheless, more studies with a bigger sample (and in other sports contexts) should be conducted to validate these conclusions. Notably, locomotor proficiencies exhibit greater stability, showing no discernible variances among adolescents with differing sports backgrounds. Taken together, these findings intimate that MC is linked to an individual’s motor experiences and motivations, rather than being directly contingent on sport-specific practice. Nonetheless, a comprehensive discourse concerning the delineation and significance of fundamental motor skills and, by extension, the constructs of MC, is imperative. Similarly, the need for longitudinal studies becomes evident as they hold the potential to provide a better understanding of these dynamics.
